# 1-(2,4-Di­nitro­phen­yl)-2-[(*E*)-2,4,5-tri­meth­oxy­benzyl­idene]hydrazine

**DOI:** 10.1107/S1600536813018345

**Published:** 2013-07-06

**Authors:** Hoong-Kun Fun, Suchada Chantrapromma, Boonlerd Nilwanna, Thawanrat Kobkeatthawin, Nawong Boonnak

**Affiliations:** a X-ray Crystallography Unit, School of Physics, Universiti Sains Malaysia, 11800 USM, Penang, Malaysia; b Department of Chemistry, Faculty of Science, Prince of Songkla University, Hat-Yai, Songkhla 90112, Thailand; c Faculty of Traditional Thai Medicine, Prince of Songkla University, Hat-Yai, Songkhla 90112, Thailand

## Abstract

The title compound, C_16_H_16_N_4_O_7_, is close to being planar, with a dihedral angle of 3.15 (11)° between the benzene rings. The meth­oxy groups at the *ortho*- and *para*-positions of the 2,4,5-tri­meth­oxy­phenyl group are almost coplanar with the ring [deviations of the C atoms = 0.017 (2) and −0.025 (2) Å, respectively], whereas the *meta*-meth­oxy group deviates slightly [C-atom displacement = 0.162 (2) Å]. Both the *ortho*- and *para*-nitro groups are close to being coplanar with their attached ring [dihedral angles = 7.81 (12) and 8.56 (11)°, respectively]. An intra­molecular N—H⋯O hydrogen bond generates an *S*(6) ring motif. In the crystal, inversion dimers linked by pairs of N—H⋯O hydrogen bonds involving the same H atom as the intra­molecular bond generate *R*_2_^2^(12) loops. The dimers are linked by weak C—H⋯O inter­actions into sheets parallel to the (10-4) plane and the sheets are stacked by π–π inter­actions, with a centroid–centroid distance of 3.5974 (14) Å.

## Related literature

For related structures, see: Fun *et al.* (2011 ▶, 2012 ▶). For background to the biological activity of hydro­zones, see: Angelusiu *et al.* (2010 ▶); Cui *et al.* (2010 ▶); Gokce *et al.* (2009 ▶); Molyneux (2004 ▶); Török *et al.* (2013 ▶); Wang *et al.* (2009 ▶).
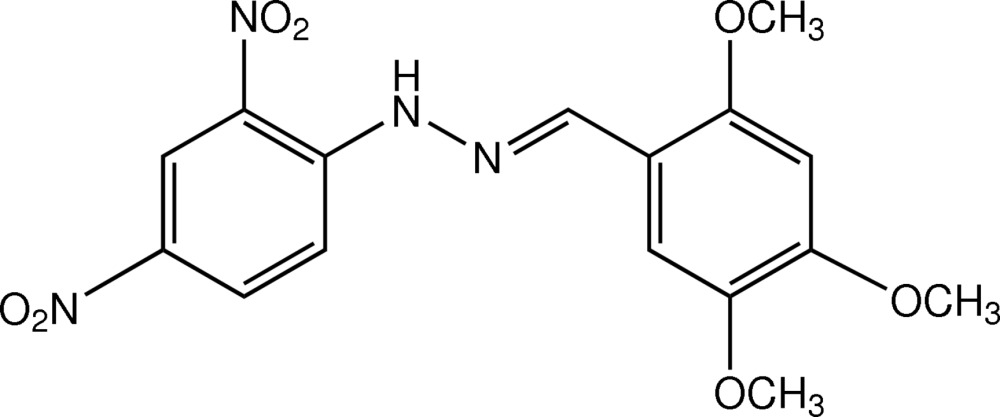


## Experimental

### 

#### Crystal data

C_16_H_16_N_4_O_7_
*M* *_r_* = 376.33
Monoclinic, 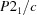

*a* = 8.0273 (13) Å
*b* = 15.048 (2) Å
*c* = 13.686 (2) Å
β = 101.546 (3)°
*V* = 1619.7 (4) Å^3^
*Z* = 4
Mo *K*α radiation
μ = 0.12 mm^−1^
*T* = 100 K
0.33 × 0.06 × 0.05 mm

#### Data collection

Bruker SMART APEXII DUO CCD diffractometer
Absorption correction: multi-scan (*SADABS*; Bruker, 2009 ▶) *T*_min_ = 0.961, *T*_max_ = 0.994
14507 measured reflections
4296 independent reflections
2385 reflections with *I* > 2σ(*I*)
*R*_int_ = 0.072

#### Refinement

*R*[*F* ^2^ > 2σ(*F* ^2^)] = 0.055
*wR*(*F* ^2^) = 0.144
*S* = 1.01
4296 reflections
251 parameters
H atoms treated by a mixture of independent and constrained refinement
Δρ_max_ = 0.31 e Å^−3^
Δρ_min_ = −0.33 e Å^−3^

### 

Data collection: *APEX2* (Bruker, 2009 ▶); cell refinement: *SAINT* (Bruker, 2009 ▶); data reduction: *SAINT*; program(s) used to solve structure: *SHELXTL* (Sheldrick, 2008 ▶); program(s) used to refine structure: *SHELXTL*; molecular graphics: *SHELXTL*; software used to prepare material for publication: *SHELXTL*, *PLATON* (Spek, 2009 ▶) and *publCIF* (Westrip, 2010 ▶).

## Supplementary Material

Crystal structure: contains datablock(s) global, I. DOI: 10.1107/S1600536813018345/hb7102sup1.cif

Structure factors: contains datablock(s) I. DOI: 10.1107/S1600536813018345/hb7102Isup2.hkl

Supplementary material file. DOI: 10.1107/S1600536813018345/hb7102Isup3.cml

Additional supplementary materials:  crystallographic information; 3D view; checkCIF report

## Figures and Tables

**Table 1:** Hydrogen-bond geometry (Å, °)

*D*—H⋯*A*	*D*—H	H⋯*A*	*D*⋯*A*	*D*—H⋯*A*
N1—H1*N*1⋯O1	0.89 (3)	2.04 (3)	2.642 (3)	124 (3)
N1—H1*N*1⋯O1^i^	0.89 (3)	2.43 (3)	3.295 (3)	164 (3)
C14—H14*A*⋯O6^ii^	0.96	2.59	3.180 (3)	120
C16—H16*C*⋯O2^iii^	0.96	2.53	3.143 (3)	122

Symmetry codes: (i) 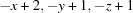; (ii) 
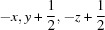
; (iii) 
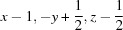
.

**Fig. 1.**
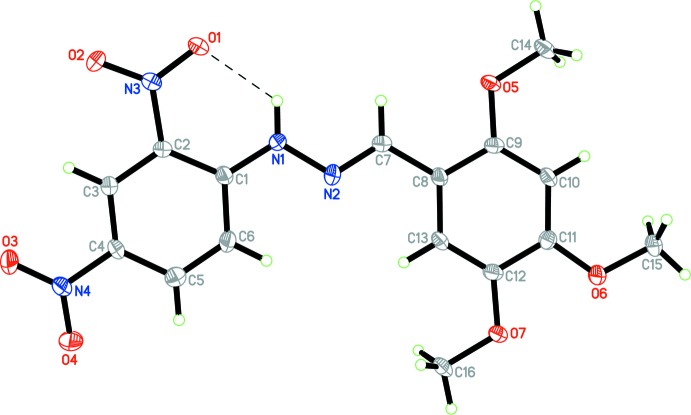
The molecular structure of (I), showing 60% probability displacement ellipsoids. The intramolecular N—H···O hydrogen bond is shown as a dashed line.

**Fig. 2.**
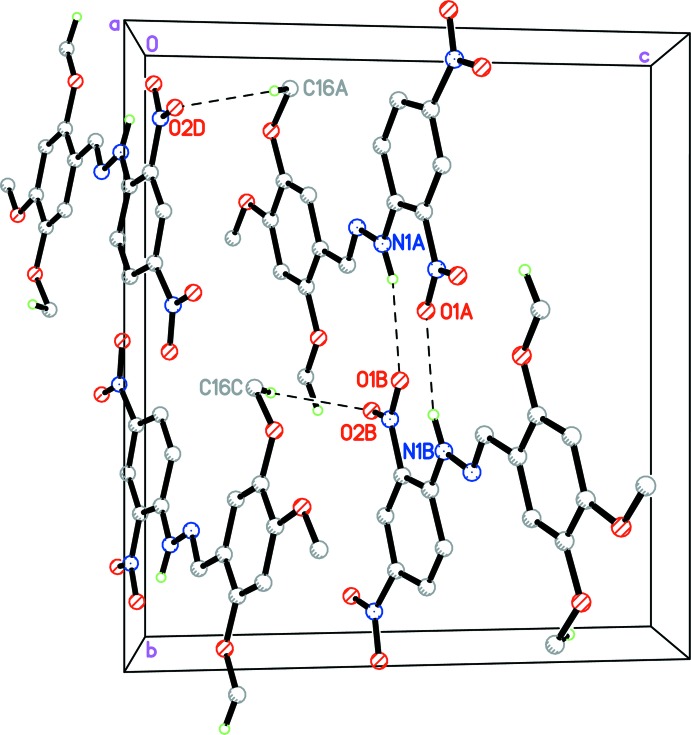
The crystal packing of (I) viewed approximately along the a axis. Hydrogen bonds are shown as dashed lines. Only H atoms involved in hydrogen bonds are shown for clarity.

**Fig. 3.**
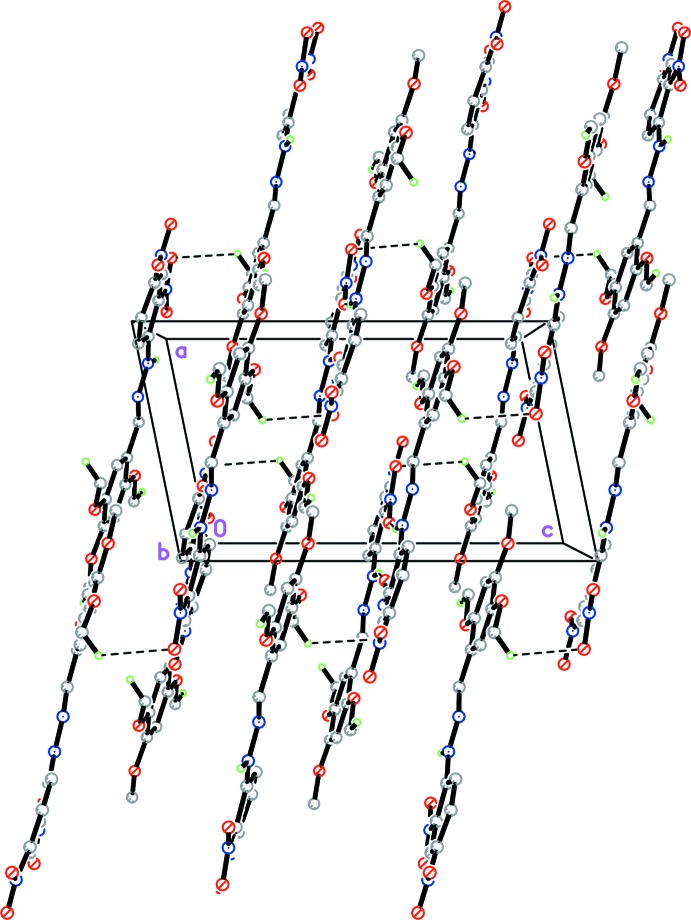
The crystal packing of (I) viewed approximately along the b axis, showing the stacking of sheets. Hydrogen bonds are shown as dashed lines. Only H atoms involved in hydrogen bonds are shown for clarity.

## supplementary crystallographic information

### Comment

Hydrazones are known to be bioactive compounds with various biological properties such as antibacterial, antifungal, antitumor, anti-inflammatory and antioxidant activities (Angelusiu *et al.*, 2010; Cui *et al.*, 2010; Gokce *et al.*, 2009 and Wang *et al.*, 2009). Diaryl hydrazones were reported to be multifunctional inhibitors of amyloid self-assembly which is related to aging-related diseases such as Alzheimer's disease (Török *et al.*, 2013). With our ongoing research on bioactive diaryl hydrazones, the title compound (I) was synthesized in order to study and compare its biological activity with the other related compounds (Fun *et al.*, 2011; 2012). Our antioxidant activity evaluation of (I) by DPPH scavenging (Molyneux, 2004) found that (I) possesses antioxidant activity with 89.04% inhibition. Furthermore its anti-Alzheimer activity is under investigation and will be reported elsewhere. Herein we report the synthesis and crystal structure of (I).

In Fig. 1, the molecular structure of (I), C_16_H_16_N_4_O_7_, is essentially planar with the dihedral angle between the two substituted benzene rings being 3.15 (11)°. Both nitro groups are slightly deviated with respect to their attached benzene rings [torsion angles O1–N3–C2–C1 = 5.4 (3)°, O2–N3–C2–C3 = 7.3 (3)°, O3–N4–C4–C3 = -5.7 (4)° and O4–N4–C4–C5 = -5.6 (3)°]. Two substituted methoxy groups at *ortho* and *para* positions of the 2,4,5-trimethoxyphenyl unit are co-planar with the bound benzene ring with the torsion angles C14–O5–C9–C10 = 1.0 (4)° and C15–O6–C11–C12 = 179.6 (2)° whereas the one at the *meta* position is slightly twisted with the torsion angle C16–O7–C12–C13 = 8.0 (4)° to reduce the steric effect. Intramolecular N1—H1N1···O1 hydrogen bond (Fig. 1 and Table 1) generates an S(6) ring motif. Bond distances in (I) are comparable with those observed in related structures (Fun *et al.*, 2011, 2012).

In the crystal packing (Fig. 2), the molecules are linked into inversion dimers by pairs of intermolecular N—H···O hydrogen bonds involving the same H atom as the intramolecular bond, generating R_2_^2^(12) loops. These dimers are then linked by weak C—H···O interactions (Table 1) into sheets parallel to the (1 0 4) plane. These sheets are further stacked (Fig. 3) by π–π interactions with distances of Cg_1_···Cg_2_^iv, v^ = 3.5974 (14) Å [symmetry codes (iv) = -1+x, y, z and (v) = 1+x, y, z].

### Experimental

The title compound (I) was synthesized by dissolving 2,4-dinitrophenylhydrazine (0.40 g, 2 mmol) in ethanol (10.00 ml) and H_2_SO_4_ (conc.) (0.50 ml) was slowly added with stirring. The solution of 2,4,5-trimethoxybenzaldehyde (0.40 g, 2 mmol) in ethanol (20.00 ml) was then added to the solution with continuous stirring for 1 hr, yielding a red solid which was filtered off and washed with methanol. Red needles of the title compound were recrystalized from ethanol solution by slow evaporation of the solvent at room temperature over several days, Mp. 528-529 K.

### Refinement

The hydrazine H atom was located from a difference Fourier map and refined isotropically. The remaining H atoms were positioned geometrically and allowed to ride on their parent atoms, with d(C-H) = 0.93 Å for CH and aromatic, and 0.96 Å for CH_3_ atoms. The *U*_iso_ values were constrained to be 1.5*U*_eq_ of the carrier atom for methyl H atoms and 1.2*U*_eq_ for the remaining H atoms. A rotating group model was used for the methyl groups.

### Crystal data

**Table:**

C_16_H_16_N_4_O_7_	*F*(000) = 784
*M**_r_* = 376.33	*D*_x_ = 1.543 Mg m^−^^3^
Monoclinic, *P*2_1_/*c*	Melting point = 528–529 K
Hall symbol: -P 2ybc	Mo *K*α radiation, λ = 0.71073 Å
*a* = 8.0273 (13) Å	Cell parameters from 4296 reflections
*b* = 15.048 (2) Å	θ = 2.0–29.0°
*c* = 13.686 (2) Å	µ = 0.12 mm^−^^1^
β = 101.546 (3)°	*T* = 100 K
*V* = 1619.7 (4) Å^3^	Needle, red
*Z* = 4	0.33 × 0.06 × 0.05 mm

### Data collection

**Table:**

Bruker SMART APEXII DUO CCD diffractometer	4296 independent reflections
Radiation source: fine-focus sealed tube	2385 reflections with *I* > 2σ(*I*)
Graphite monochromator	*R*_int_ = 0.072
φ and ω scans	θ_max_ = 29.0°, θ_min_ = 2.0°
Absorption correction: multi-scan (*SADABS*; Bruker, 2009)	*h* = −10→10
*T*_min_ = 0.961, *T*_max_ = 0.994	*k* = −17→20
14507 measured reflections	*l* = −18→18

### Refinement

**Table:**

Refinement on *F*^2^	Primary atom site location: structure-invariant direct methods
Least-squares matrix: full	Secondary atom site location: difference Fourier map
*R*[*F*^2^ > 2σ(*F*^2^)] = 0.055	Hydrogen site location: inferred from neighbouring sites
*wR*(*F*^2^) = 0.144	H atoms treated by a mixture of independent and constrained refinement
*S* = 1.01	*w* = 1/[σ^2^(*F*_o_^2^) + (0.0544*P*)^2^ + 0.5503*P*] where *P* = (*F*_o_^2^ + 2*F*_c_^2^)/3
4296 reflections	(Δ/σ)_max_ < 0.001
251 parameters	Δρ_max_ = 0.31 e Å^−^^3^
0 restraints	Δρ_min_ = −0.33 e Å^−^^3^

### Special details

**Table:**

Experimental. The crystal was placed in the cold stream of an Oxford Cryosystems Cobra open-flow nitrogen cryostat (Cosier & Glazer, 1986) operating at 100.0 (1) K.
Geometry. All esds (except the esd in the dihedral angle between two l.s. planes) are estimated using the full covariance matrix. The cell esds are taken into account individually in the estimation of esds in distances, angles and torsion angles; correlations between esds in cell parameters are only used when they are defined by crystal symmetry. An approximate (isotropic) treatment of cell esds is used for estimating esds involving l.s. planes.
Refinement. Refinement of F^2^ against ALL reflections. The weighted R-factor wR and goodness of fit S are based on F^2^, conventional R-factors R are based on F, with F set to zero for negative F^2^. The threshold expression of F^2^ > 2sigma(F^2^) is used only for calculating R-factors(gt) etc. and is not relevant to the choice of reflections for refinement. R-factors based on F^2^ are statistically about twice as large as those based on F, and R- factors based on ALL data will be even larger.

### Fractional atomic coordinates and isotropic or equivalent isotropic displacement parameters (Å^2^)

**Table:**

	*x*	*y*	*z*	*U*_iso_*/*U*_eq_	
O1	1.12652 (19)	0.44523 (11)	0.52056 (12)	0.0210 (4)	
O2	1.38293 (19)	0.39446 (11)	0.56829 (12)	0.0204 (4)	
O3	1.4973 (2)	0.08730 (12)	0.60481 (14)	0.0302 (5)	
O4	1.2974 (2)	−0.00920 (12)	0.55543 (14)	0.0292 (5)	
O5	0.33101 (19)	0.48609 (11)	0.33462 (13)	0.0226 (4)	
O6	−0.05504 (19)	0.24861 (11)	0.19418 (12)	0.0201 (4)	
O7	0.18289 (19)	0.13357 (11)	0.24418 (12)	0.0187 (4)	
N1	0.8715 (2)	0.33762 (14)	0.44831 (15)	0.0166 (5)	
H1N1	0.891 (4)	0.394 (2)	0.466 (2)	0.041 (9)*	
N2	0.7106 (2)	0.30856 (14)	0.40437 (14)	0.0171 (5)	
N3	1.2304 (2)	0.38263 (13)	0.53664 (14)	0.0164 (5)	
N4	1.3513 (2)	0.06807 (14)	0.56381 (15)	0.0194 (5)	
C1	0.9955 (3)	0.27522 (16)	0.46967 (16)	0.0142 (5)	
C2	1.1676 (3)	0.29321 (15)	0.51555 (16)	0.0149 (5)	
C3	1.2840 (3)	0.22554 (16)	0.54419 (16)	0.0153 (5)	
H3A	1.3951	0.2386	0.5756	0.018*	
C4	1.2341 (3)	0.13928 (16)	0.52577 (17)	0.0162 (5)	
C5	1.0706 (3)	0.11895 (17)	0.47365 (17)	0.0182 (6)	
H5A	1.0407	0.0602	0.4579	0.022*	
C6	0.9552 (3)	0.18490 (16)	0.44595 (16)	0.0169 (5)	
H6A	0.8472	0.1705	0.4106	0.020*	
C7	0.5914 (3)	0.36708 (16)	0.38720 (16)	0.0163 (5)	
H7A	0.6128	0.4261	0.4056	0.020*	
C8	0.4214 (3)	0.33803 (16)	0.33783 (16)	0.0144 (5)	
C9	0.2900 (3)	0.39983 (16)	0.31141 (17)	0.0157 (5)	
C10	0.1277 (3)	0.37208 (16)	0.26307 (17)	0.0163 (5)	
H10A	0.0409	0.4135	0.2453	0.020*	
C11	0.0970 (3)	0.28289 (16)	0.24183 (16)	0.0151 (5)	
C12	0.2279 (3)	0.21986 (16)	0.26910 (16)	0.0155 (5)	
C13	0.3866 (3)	0.24807 (16)	0.31568 (16)	0.0158 (5)	
H13A	0.4733	0.2065	0.3330	0.019*	
C14	0.2019 (3)	0.55210 (17)	0.3101 (2)	0.0251 (6)	
H14A	0.2473	0.6090	0.3333	0.038*	
H14B	0.1084	0.5377	0.3414	0.038*	
H14C	0.1629	0.5540	0.2391	0.038*	
C15	−0.1945 (3)	0.30917 (17)	0.16478 (18)	0.0212 (6)	
H15A	−0.2911	0.2775	0.1284	0.032*	
H15B	−0.1623	0.3549	0.1232	0.032*	
H15C	−0.2233	0.3356	0.2231	0.032*	
C16	0.3069 (3)	0.06688 (16)	0.28081 (18)	0.0208 (6)	
H16A	0.2600	0.0092	0.2623	0.031*	
H16B	0.3380	0.0709	0.3521	0.031*	
H16C	0.4058	0.0759	0.2525	0.031*	

### Atomic displacement parameters (Å^2^)

**Table:**

	*U*^11^	*U*^22^	*U*^33^	*U*^12^	*U*^13^	*U*^23^
O1	0.0171 (8)	0.0103 (9)	0.0338 (9)	0.0024 (7)	0.0009 (7)	−0.0002 (7)
O2	0.0122 (8)	0.0163 (9)	0.0305 (9)	−0.0031 (7)	−0.0009 (7)	−0.0019 (7)
O3	0.0140 (8)	0.0199 (10)	0.0520 (11)	0.0024 (7)	−0.0048 (8)	0.0026 (9)
O4	0.0239 (9)	0.0111 (9)	0.0488 (11)	−0.0020 (7)	−0.0020 (8)	0.0018 (8)
O5	0.0148 (8)	0.0091 (9)	0.0412 (10)	0.0022 (7)	−0.0008 (8)	−0.0012 (8)
O6	0.0118 (8)	0.0155 (9)	0.0305 (9)	0.0012 (7)	−0.0021 (7)	−0.0011 (7)
O7	0.0148 (8)	0.0106 (9)	0.0286 (9)	0.0013 (7)	−0.0007 (7)	−0.0019 (7)
N1	0.0112 (9)	0.0111 (11)	0.0257 (10)	−0.0010 (8)	−0.0004 (8)	0.0006 (9)
N2	0.0099 (9)	0.0170 (11)	0.0232 (9)	0.0000 (8)	0.0001 (8)	−0.0001 (8)
N3	0.0161 (9)	0.0110 (10)	0.0211 (10)	0.0015 (8)	0.0017 (8)	−0.0002 (8)
N4	0.0156 (10)	0.0123 (11)	0.0301 (11)	0.0019 (8)	0.0039 (9)	0.0005 (9)
C1	0.0118 (10)	0.0128 (12)	0.0180 (10)	0.0013 (9)	0.0032 (9)	0.0007 (9)
C2	0.0138 (10)	0.0102 (12)	0.0198 (10)	−0.0005 (9)	0.0016 (9)	−0.0008 (9)
C3	0.0110 (10)	0.0139 (12)	0.0204 (11)	0.0008 (9)	0.0014 (9)	−0.0006 (9)
C4	0.0131 (11)	0.0128 (12)	0.0216 (11)	0.0039 (9)	0.0012 (9)	0.0010 (10)
C5	0.0166 (11)	0.0157 (13)	0.0223 (12)	−0.0014 (10)	0.0035 (10)	−0.0005 (10)
C6	0.0107 (10)	0.0177 (13)	0.0212 (11)	−0.0006 (9)	0.0004 (9)	0.0011 (10)
C7	0.0153 (11)	0.0112 (12)	0.0215 (11)	−0.0009 (9)	0.0018 (9)	−0.0002 (9)
C8	0.0102 (10)	0.0125 (12)	0.0203 (11)	0.0012 (9)	0.0027 (9)	0.0013 (9)
C9	0.0153 (11)	0.0100 (12)	0.0213 (11)	−0.0011 (9)	0.0024 (9)	−0.0007 (9)
C10	0.0133 (11)	0.0125 (12)	0.0222 (11)	0.0040 (9)	0.0013 (9)	0.0017 (10)
C11	0.0104 (10)	0.0148 (13)	0.0199 (11)	−0.0008 (9)	0.0025 (9)	0.0000 (9)
C12	0.0160 (11)	0.0107 (12)	0.0198 (11)	−0.0001 (9)	0.0035 (9)	−0.0008 (9)
C13	0.0133 (11)	0.0115 (12)	0.0220 (11)	0.0041 (9)	0.0019 (9)	0.0005 (9)
C14	0.0203 (12)	0.0119 (13)	0.0408 (14)	0.0062 (10)	0.0006 (11)	0.0003 (11)
C15	0.0122 (11)	0.0199 (14)	0.0292 (12)	0.0048 (10)	−0.0014 (10)	0.0000 (11)
C16	0.0177 (12)	0.0122 (13)	0.0312 (12)	0.0049 (10)	0.0018 (10)	0.0015 (10)

### Geometric parameters (Å, º)

**Table:**

O1—N3	1.248 (2)	C5—C6	1.359 (3)
O2—N3	1.227 (2)	C5—H5A	0.9300
O3—N4	1.228 (2)	C6—H6A	0.9300
O4—N4	1.238 (3)	C7—C8	1.463 (3)
O5—C9	1.361 (3)	C7—H7A	0.9300
O5—C14	1.427 (3)	C8—C9	1.398 (3)
O6—C11	1.364 (2)	C8—C13	1.403 (3)
O6—C15	1.437 (3)	C9—C10	1.401 (3)
O7—C12	1.372 (3)	C10—C11	1.385 (3)
O7—C16	1.432 (3)	C10—H10A	0.9300
N1—C1	1.357 (3)	C11—C12	1.410 (3)
N1—N2	1.382 (2)	C12—C13	1.373 (3)
N1—H1N1	0.88 (3)	C13—H13A	0.9300
N2—C7	1.287 (3)	C14—H14A	0.9600
N3—C2	1.446 (3)	C14—H14B	0.9600
N4—C4	1.452 (3)	C14—H14C	0.9600
C1—C6	1.420 (3)	C15—H15A	0.9600
C1—C2	1.424 (3)	C15—H15B	0.9600
C2—C3	1.385 (3)	C15—H15C	0.9600
C3—C4	1.367 (3)	C16—H16A	0.9600
C3—H3A	0.9300	C16—H16B	0.9600
C4—C5	1.396 (3)	C16—H16C	0.9600
			
C9—O5—C14	118.60 (17)	C9—C8—C7	120.4 (2)
C11—O6—C15	117.78 (18)	C13—C8—C7	121.0 (2)
C12—O7—C16	116.49 (16)	O5—C9—C8	116.05 (18)
C1—N1—N2	117.2 (2)	O5—C9—C10	123.6 (2)
C1—N1—H1N1	121.4 (19)	C8—C9—C10	120.4 (2)
N2—N1—H1N1	121.2 (19)	C11—C10—C9	119.8 (2)
C7—N2—N1	117.3 (2)	C11—C10—H10A	120.1
O2—N3—O1	122.47 (19)	C9—C10—H10A	120.1
O2—N3—C2	119.27 (19)	O6—C11—C10	124.9 (2)
O1—N3—C2	118.25 (17)	O6—C11—C12	114.9 (2)
O3—N4—O4	123.2 (2)	C10—C11—C12	120.29 (19)
O3—N4—C4	118.7 (2)	O7—C12—C13	125.6 (2)
O4—N4—C4	118.03 (17)	O7—C12—C11	115.18 (18)
N1—C1—C6	119.28 (19)	C13—C12—C11	119.2 (2)
N1—C1—C2	124.6 (2)	C12—C13—C8	121.6 (2)
C6—C1—C2	116.1 (2)	C12—C13—H13A	119.2
C3—C2—C1	121.7 (2)	C8—C13—H13A	119.2
C3—C2—N3	116.05 (18)	O5—C14—H14A	109.5
C1—C2—N3	122.3 (2)	O5—C14—H14B	109.5
C4—C3—C2	119.31 (19)	H14A—C14—H14B	109.5
C4—C3—H3A	120.3	O5—C14—H14C	109.5
C2—C3—H3A	120.3	H14A—C14—H14C	109.5
C3—C4—C5	120.9 (2)	H14B—C14—H14C	109.5
C3—C4—N4	119.29 (18)	O6—C15—H15A	109.5
C5—C4—N4	119.8 (2)	O6—C15—H15B	109.5
C6—C5—C4	120.1 (2)	H15A—C15—H15B	109.5
C6—C5—H5A	120.0	O6—C15—H15C	109.5
C4—C5—H5A	120.0	H15A—C15—H15C	109.5
C5—C6—C1	121.61 (19)	H15B—C15—H15C	109.5
C5—C6—H6A	119.2	O7—C16—H16A	109.5
C1—C6—H6A	119.2	O7—C16—H16B	109.5
N2—C7—C8	118.1 (2)	H16A—C16—H16B	109.5
N2—C7—H7A	121.0	O7—C16—H16C	109.5
C8—C7—H7A	121.0	H16A—C16—H16C	109.5
C9—C8—C13	118.60 (19)	H16B—C16—H16C	109.5
			
C1—N1—N2—C7	177.6 (2)	N2—C7—C8—C9	−175.7 (2)
N2—N1—C1—C6	0.3 (3)	N2—C7—C8—C13	4.0 (4)
N2—N1—C1—C2	−179.5 (2)	C14—O5—C9—C8	−179.6 (2)
N1—C1—C2—C3	173.6 (2)	C14—O5—C9—C10	1.0 (4)
C6—C1—C2—C3	−6.2 (4)	C13—C8—C9—O5	−179.9 (2)
N1—C1—C2—N3	−5.3 (4)	C7—C8—C9—O5	−0.2 (3)
C6—C1—C2—N3	174.9 (2)	C13—C8—C9—C10	−0.5 (4)
O2—N3—C2—C3	7.3 (3)	C7—C8—C9—C10	179.2 (2)
O1—N3—C2—C3	−173.5 (2)	O5—C9—C10—C11	179.7 (2)
O2—N3—C2—C1	−173.7 (2)	C8—C9—C10—C11	0.4 (4)
O1—N3—C2—C1	5.4 (3)	C15—O6—C11—C10	−0.8 (3)
C1—C2—C3—C4	1.7 (4)	C15—O6—C11—C12	179.6 (2)
N3—C2—C3—C4	−179.4 (2)	C9—C10—C11—O6	−179.2 (2)
C2—C3—C4—C5	3.6 (4)	C9—C10—C11—C12	0.3 (4)
C2—C3—C4—N4	−174.4 (2)	C16—O7—C12—C13	8.0 (4)
O3—N4—C4—C3	−5.7 (4)	C16—O7—C12—C11	−173.1 (2)
O4—N4—C4—C3	172.5 (2)	O6—C11—C12—O7	−0.3 (3)
O3—N4—C4—C5	176.2 (2)	C10—C11—C12—O7	−179.9 (2)
O4—N4—C4—C5	−5.6 (3)	O6—C11—C12—C13	178.7 (2)
C3—C4—C5—C6	−4.2 (4)	C10—C11—C12—C13	−0.9 (4)
N4—C4—C5—C6	173.9 (2)	O7—C12—C13—C8	179.6 (2)
C4—C5—C6—C1	−0.8 (4)	C11—C12—C13—C8	0.7 (4)
N1—C1—C6—C5	−174.1 (2)	C9—C8—C13—C12	0.0 (4)
C2—C1—C6—C5	5.7 (3)	C7—C8—C13—C12	−179.8 (2)
N1—N2—C7—C8	178.1 (2)		

### Hydrogen-bond geometry (Å, º)

**Table:**

*D*—H···*A*	*D*—H	H···*A*	*D*···*A*	*D*—H···*A*
N1—H1*N*1···O1	0.89 (3)	2.04 (3)	2.642 (3)	124 (3)
N1—H1*N*1···O1^i^	0.89 (3)	2.43 (3)	3.295 (3)	164 (3)
C14—H14*A*···O6^ii^	0.96	2.59	3.180 (3)	120
C16—H16*C*···O2^iii^	0.96	2.53	3.143 (3)	122

Symmetry codes: (i) −*x*+2, −*y*+1, −*z*+1; (ii) −*x*, *y*+1/2, −*z*+1/2; (iii) *x*−1, −*y*+1/2, *z*−1/2.

## References

[bb1] Angelusiu, M.-V., Barbuceanu, S.-F., Draghici, C. & Almajan, G.-L. (2010). *Eur. J. Med. Chem* **45**, 2055–2062. 10.1016/j.ejmech.2010.01.033 20133023

[bb2] Bruker (2009). *APEX2*, *SAINT* and *SADABS* Bruker AXS Inc., Madison, Wisconsin, USA.

[bb3] Cui, Z., Li, Y., Ling, Y., Huang, J., Cui, J., Wang, R. & Yang, X. (2010). *Eur. J. Med. Chem.* **45**, 5576–5584. 10.1016/j.ejmech.2010.09.007 20884091

[bb4] Fun, H.-K., Chantrapromma, S., Nilwanna, B. & Kobkeatthawin, T. (2012). *Acta Cryst.* E**68**, o2144–o2145. 10.1107/S1600536812026979 PMC3393954 22798819

[bb5] Fun, H.-K., Nilwanna, B., Jansrisewangwong, P., Kobkeatthawin, T. & Chantrapromma, S. (2011). *Acta Cryst.* E**67**, o3202–o3203. 10.1107/S1600536811045417 PMC3238869 22199722

[bb6] Gokce, M., Utku, S. & Kupeli, E. (2009). *Eur. J. Med. Chem.* **44**, 3760–3764. 10.1016/j.ejmech.2009.04.048 19535179

[bb7] Molyneux, P. (2004). *Songklanakarin J. Sci. Technol* **26**, 211–219.

[bb8] Sheldrick, G. M. (2008). *Acta Cryst.* A**64**, 112–122. 10.1107/S0108767307043930 18156677

[bb9] Spek, A. L. (2009). *Acta Cryst.* D**65**, 148–155. 10.1107/S090744490804362X PMC2631630 19171970

[bb10] Török, B., Sood, A., Bag, S., Tulsan, R., Ghosh, S., Borkin, D., Kennedy, A. R., Melanson, M., Madden, R., Zhou, W., LeVine, H. & Török, M. (2013). *Biochemistry*, **52**, 1137–1148. 10.1021/bi3012059 PMC3608202 23346953

[bb11] Wang, Q., Yang, Z. Y., Qi, G.-F. & Qin, D.-D. (2009). *Eur. J. Med. Chem.* **44**, 2425–2433. 10.1016/j.ejmech.2008.10.023 19038478

[bb12] Westrip, S. P. (2010). *J. Appl. Cryst.* **43**, 920–925.

